# The Role of ERα36 in Development and Tumor Malignancy

**DOI:** 10.3390/ijms21114116

**Published:** 2020-06-09

**Authors:** Charlène Thiebaut, Henri-Philippe Konan, Marie-Justine Guerquin, Amand Chesnel, Gabriel Livera, Muriel Le Romancer, Hélène Dumond

**Affiliations:** 1 Université de Lorraine, CNRS, CRAN, F-54000 Nancy, France; charlene.thiebaut@univ-lorraine.fr (C.T.); amand.chesnel@hotmail.fr (A.C.); 2 Université de Lyon, F-69000 Lyon, France; HenriPhilippe.KONAN@lyon.unicancer.fr (H.-P.K.); Muriel.LEROMANCER-CHERIFI@lyon.unicancer.fr (M.L.R.); 3 INSERM U1052, Centre de Recherche en Cancérologie de Lyon, F-69000 Lyon, France; 4 CNRS UMR5286, Centre de Recherche en Cancérologie de Lyon, F-69000 Lyon, France; 5 Laboratory of Development of the Gonads, UMRE008 Genetic Stability Stem Cells and Radiation, Université de Paris, Université Paris Saclay, CEA, F-92265 Fontenay aux Roses, France; marie-justine.guerquin@cea.fr (M.-J.G.); gabriel.livera@cea.fr (G.L.)

**Keywords:** ERα36, estrogen signaling, breast cancer, endocrine therapy resistance

## Abstract

Estrogen nuclear receptors, represented by the canonical forms ERα66 and ERβ1, are the main mediators of the estrogen-dependent pathophysiology in mammals. However, numerous isoforms have been identified, stimulating unconventional estrogen response pathways leading to complex cellular and tissue responses. The estrogen receptor variant, ERα36, was cloned in 2005 and is mainly described in the literature to be involved in the progression of mammary tumors and in the acquired resistance to anti-estrogen drugs, such as tamoxifen. In this review, we will first specify the place that ERα36 currently occupies within the diversity of nuclear and membrane estrogen receptors. We will then report recent data on the impact of ERα36 expression and/or activity in normal breast and testicular cells, but also in different types of tumors including mammary tumors, highlighting why ERα36 can now be considered as a marker of malignancy. Finally, we will explain how studying the regulation of ERα36 expression could provide new clues to counteract resistance to cancer treatments in hormone-sensitive tumors.

## 1. Introduction

The term “estrogen” refers to a set of four steroid hormones naturally produced by the body: estrone (E1), 17β-estradiol (E2), estriol (E3), and estetrol (E4). Like all steroid hormones, estrogens are synthesized from cholesterol (27 carbons) via an enzymatic biosynthetic pathway, which takes place mainly in the ovaries in premenopausal women and in the placenta during pregnancy. When the ovarian function ceases at the time of menopause, fewer estrogens are produced by peripheral tissues, such as the adrenal glands, the liver, the brain, and adipose tissue. Estrogens are involved in the regulation of a large number of pathophysiological processes in women, but also in men. These hormones act to ensure and regulate the reproductive system, but also the neuroendocrine, cardiovascular, immune, and skeletal systems [1].

Estrogen effects are primarily mediated by the nuclear estrogen receptors ERα and ERβ. ERα was cloned in 1986 from a cDNA library derived from MCF-7 breast cancer cells [2] and ERβ was cloned ten years later from a cDNA library derived from rat prostate [3]. These receptors belong to the NR3 class of nuclear receptors and are encoded respectively by the *ESR1* gene located on chromosome 6 (6q25.1) and the *ESR2* gene located on chromosome 14 (14q23.2) [4,5]. In addition to the canonical forms ERα66 and ERβ1, different splicing and promoter variants have been cloned. These receptors are transcription factors able of directly regulating the expression of their target genes and are therefore involved in the genomic estrogen signaling.

In parallel, estrogen can also activate a non-genomic estrogen signaling which is initiated from estrogen receptors located at the membrane level. In 1997, Carmeci and colleagues [6] cloned GPR30 from the breast cancer cell line MCF-7, further demonstrated by Revankar and colleagues [7] to be a membrane estrogen receptor belonging to the superfamily of receptors coupled to G proteins. This receptor called GPR30 or GPER (G-protein-coupled estrogen receptor) is encoded by the *GPER1* gene. Regarding its location, GPER is mainly found at the plasma membrane and the endoplasmic reticulum membrane [7]. Initially, GPER was considered as an orphan receptor but in 2005, Thomas and colleagues [8] demonstrated that estrogens are able to interact with GPER, which can also bind phytoestrogens like Genistein as well as xenoestrogens like Bisphenol A.

Interestingly, it has also been shown that different variants of nuclear estrogen receptors alpha and beta can be found at the plasma membrane following post-translational modifications, such as palmitoylation or myristoylation. This is notably the case for ERα66, ERβ1, ERα36, and ERα46 [9,10,11,12]. This sub-membrane localization is associated with the activation of non-genomic estrogen signaling.

In this review, we will focus on the estrogen receptor variant ERα36 which is primarily located at the plasma membrane and described as the main mediator of estrogen non-genomic signaling. Since its cloning, an increasing number of studies suggest that this variant plays a major role in the progression of various cancers and in the development of resistance to anti-tumor treatments. However, its expression is never taken into account in the clinical diagnosis. We will also report recent data on the role of ERα36 in the pathophysiology of hormone-sensitive organs and new strategies arising from in vitro studies aimed to modulate ERα36 expression and function in cancers.

## 2. ERα36 in the Landscape of ERα Variants

**Figure 1:**
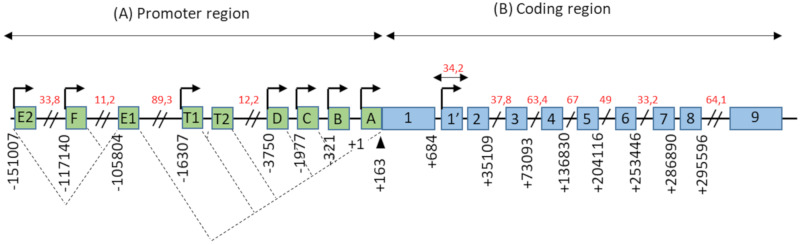
Genomic organization of the *ESR1* gene. (**A**) The promoter region of the *ESR1* gene contains 9 non-coding exons (green boxes), annotated from A to F—T1, T2, and 7 alternative promoters each having its own TSS (arrows). There is also a splice acceptor site located at position +163 bp. The dotted lines symbolize the different possibilities for the splicing of non-coding exons, as described in the literature. Exons E1 and T2 expression depends on the promoters E2 and T1, respectively. (**B**) The coding region of the *ESR1* gene consists of 10 exons (blue boxes), numbered from 1–1′to 9, separated by 9 introns. There is also an 8th alternative promoter located at the level of exon 1′. In this schematic representation, the scale is not respected but some estimated positions are indicated below the exons, in base pairs (bp). Position +1 corresponds to the first transcription initiation site (TSS) described by Greene and colleagues, in 1986 [2]. The indications in red correspond to the length of long intronic regions in kilo base pairs (kbp). Adapted from [14,15].

The promoter region of the *ESR1* gene spans more than 150 kb on chromosome 6 (6q25.1), and contains at least nine non-coding exons and seven alternative promoters which contribute to regulating the differential expression of ERα in different estrogen target tissues (Figure 1). The first transcription start site (TSS), located at the level of the non-coding exon “A”, was identified by Greene and colleagues in 1986 [2]. In addition, Keaveney and colleagues [13] demonstrated the existence of an acceptor splice site localized 163 bp downstream of the exon A TSS. This site is highly conserved among vertebrates as the point of anchorage for the various non-coding exons of the promoter region. Then, the majority of non-coding exons were shown to have their own TSS [14]. Moreover, an eighth alternative promoter located upstream of exon 1 has been described [14,15,16].

The coding region of the *ESR1* gene spans over 300 kb and consists of 10 exons and nine introns, which are the source of four isoforms: ERα66, ERα46, ERα36, and ERα30. The canonical form of ERα, called ERα66, is a 595 amino acid protein of 66 kDa. ERα66 displays all of the functional domains typical for nuclear receptors [17]. The A/B domain located at the N-terminal end is the least conserved domain between the members of the superfamily of nuclear receptors, both in terms of length and of amino acid sequence homology. This region contains the AF-1 transactivation domain that allows ligand-independent activation of transcription. In addition, the A/B domain has several phosphorylation and sumoylation sites that allow the recruitment of co-activators or co-repressors and can modulate the regulation of ERα66 target genes [18]. The C domain, also called DBD (DNA-binding domain), is a highly conserved domain which allows the attachment of ERα66 to specific response elements located in the promoter region of the target genes. This domain consists of 2 zinc finger motifs, each consisting of four cysteines surrounding a Zn^2+^ ion. At the base of the first zinc finger is a P-box (proximal box) motif involved in the recognition of the response elements, while the second zinc finger is associated with a D-box (distal box), which is necessary for ERα66 dimerization and function [19,20,21]. The D domain is a flexible hinge region that allows the rotation of the DBD to facilitate the binding of the receptor to its response element. The nuclear localization signal that allows the translocation of the receptor to the nucleus is present in this poorly conserved region. This sequence is exposed as a result of a change in the D domain conformation induced by ligand binding [22]. The E/F domain is the ligand-binding domain (LBD) and participates in the dimerization of the receptor. For most nuclear receptors, including ERα66, this E/F domain consists of 12 α helices and an antiparallel beta sheet, which form the binding pocket for hydrophobic ligands. This region also contains the AF-2 transactivation domain, which consists of the H3, H4, and H12 helices, and which results in the ligand-dependent activation of transcription. Indeed, depending on the agonist or antagonist nature of the ligand, the orientation of the H12 helix is different, and has an impact on the recruitment of the co-factors needed to regulate ERα66 transcriptional activity [23,24].

**Figure 2:**
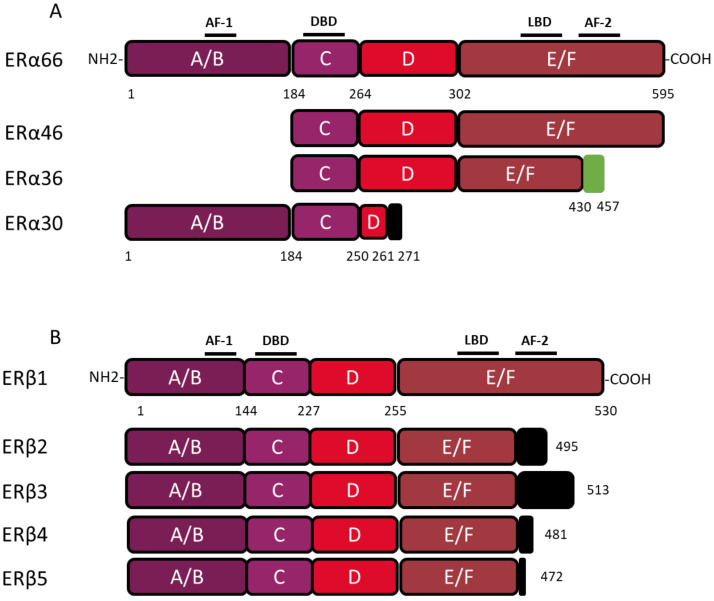
Structure of canonical forms and different variants of ERα66 and ERβ1. The canonical ERα 66 and ERβ1 respectively encompass 595 and 530 amino acids. (**A**) The ERα66 variants, ERα46 and ERα36, do not display a A/B domain which is involved in ligand-independent transactivation. In addition, the ERα36 variant has a truncated E/F domain and a small specific sequence, of 27 amino acids (green box), at the C-terminal end. The ERα30 variant retains only the A/B domain, the C domain and part of a D domain. In the C-terminus this variant has a short specific sequence of 10 amino acids (black box). (**B**) The 4 variants of ERβ, all have a truncated E/F domain and a small specific sequence of variable size at the C-terminal end (black box). Characteristic of ERβ2, the 26 amino acids specific to the C-terminal end are encoded by an exon called “exon 9” or “exon cx” located downstream of exon 8 of the *ESR2* gene. Adapted from [28,29].

The three variants of ERα66, namely ERα46, ERα36, and ERα30, result from alternative splicing mechanisms, but also from the use of alternative promoters (Figure 1). Unlike the canonical form ERα66, which is encoded by exons 1–8, the variant ERα46 is transcribed from promoters E and F and encoded from exons 2 to 8. At the protein level (Figure 2), this 46 kDa variant lacks the AF-1 transactivation domain. ERα46 can be palmitoylated by the PAT (palmitoyltransferase) enzyme and thus recruited to the caveolae at the plasma membrane [25]. This variant triggers more potent non-genomic signaling than ERα66 [10]. Moreover, ERα46 expression in ERα-positive tumors is associated with less aggressive tumors, likely due to the fact that it decreases the proliferation rate in response to E_2_ [26].

More recently, a 30 kDa variant, called ERα30, was identified by Zhu H. and colleagues [27]. This variant is encoded by exons 1, 2, 3, and 8, but also by a part of exons 4 and 6. In effect, during alternative splicing, the first 24 nucleotides of exon 4 are directly linked to the last 44 nucleotides of exon 6. At the protein level, it results in a truncated D hinge domain as well as in the absence of the LBD and the AF-2 transactivation domain. ERα30 has a small C-terminal 10 amino acid-specific region, of which the function and impact on the structure of the protein are not described [27].

The ERα36 variant is transcribed from an alternative promoter located in the first intron of the *ESR1* gene and is encoded by exons 1′, 2–6, and 9. From a functional point of view, the ERα36 protein, retains the DBD, as well as part of the LBD, but not AF-1 and AF-2 transactivation domains. ERα36 also possesses a specific C-terminal region of 27 amino acids encoded by exon 9 and containing a functional nuclear export signal, inducing the exportation of ERα36 from the nucleus [15,30]. Moreover, this unique C-terminal domain of ERα36 and more precisely the L297 residue plays a crucial role in the interaction between ERα36 and p-ERK2 [30].

## 3. Consequences of ERα36 Expression in Non-Cancerous Cells

Since its cloning in 2005, the ERα36 variant has been mainly described in the literature for its role in the progression of different types of cancer. However, the ERα36 mRNA was also found in various healthy estrogen-sensitive tissues such as the mammary gland, the uterus, the bone and cartilage tissues, renal tubules, the stomach, etc. [31,32,33,34,35,36]. This suggested that this variant could participate in mediating the physiological effects of estrogens.

### 3.1. In Vivo

#### 3.1.1. Impact of ERα36 on the Development of the Male Germ Line

Testicular germ cell tumors (TGCTs) are the most common cancers in young men. The origin of these cancers is not fully understood but genetic and cellular evidence suggest that they arise from embryonic germ cells. Fetal exposure to xenoestrogens appear to alter fetal germ cell differentiation leading to carcinogenesis [37,38]. Previous studies from Ajj and colleagues [39] demonstrated the involvement of ERα36 in the progression of TGCTs. Therefore, Guerquin and colleagues aimed at characterizing ERα36 expression in human fetal gonads by collecting male and female gonads from 6 to 18 weeks of gestation. This period covers the transition from a pluripotent state to a differentiated state. Unlike ERα66, ERα36 mRNA and protein were not detected either by quantitative PCR, Western blot or immunohistochemistry in these samples. Different fetal tissues (bone, liver, skin, intestine, brain, lung, heart, etc.) were also collected between six and 10 weeks of gestation to characterize the expression of ERα66, ERα36, and ERβ by quantitative PCR. The mRNA of ERα36 was not detected in the different tissues tested unlike ERα66 and ERβ.

The impact of this variant on the physiological development of the murine male germ line was also studied. Since the sequence encoding ERα36 is not present in the mouse genome, a C57BL/6J transgenic line specifically expressing ERα36 in the germline was developed. More precisely, the ERα36 coding sequence was cloned under the control of the promoter of Pou5f1 deleted from its proximal element (Oct4-∆PE). Indeed, unlike the distal element, which drives expression restricted to the germline, the proximal element of the Oct4 promoter can be activated in other cell types derived from the epiblast. In addition, the Oct4-∆PE promoter allows the expression of the transgene from 8 dpc (day post-coitum) in primordial germ cells [40,41]. The study of the Oct4-ERα36 transgenic model was carried out by to monitor fetal germ cell differentiation after transgene expression and the consequence on fertility in adulthood. A transcriptomic analysis was performed on male fetal germ cells collected from wild-type (wt) and transgenic animals (Tg Oct4-ERα36) at 15.5 dpc. Thereby, 1365 genes, the expression of which varies significantly (*p* < 0.05) between the wt and the Tg Oct4-ERα36 lots, were submitted to functional analyses through the “Panther (Protein ANalysis THrough Evolutionary Relationships) Classification System” databases (http://pantherdb.org/) and “DAVID (Database for Annotation, Visualization and Integrated Discovery)” (https://david.ncifcrf.gov/).

**Figure 3:**
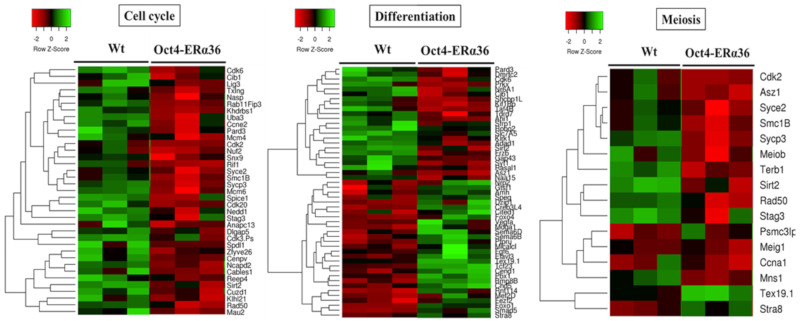
The main biological processes affected by ERα36 expression in mouse fetal testis. Heatmaps showing the expression level of differentially expressed genes involved in «Cell cycle», «Differentiation» and «Meiosis» processes in Oct4-ERα36 C57BL6/J transgenic mice compared to wild-type ones. Cell cycle: 37 genes; Differentiation: 51 genes and Meiosis: 16 genes. Red: down regulated genes vs. green: up-regulated genes. For each experimental condition, 3 biological replicats of independent fetal gonad pools are presented.

The results obtained indicated that ERα36-dependent signaling modulates the expression of genes associated with the GO terms: “cell cycle”, “meiosis”, and “differentiation” (*p*-value < 3 × 10^−2^) (Figure 3), suggesting that the expression of this variant in the male germ line is associated with a modulation of the proliferation, meiotic division, and differentiation processes which could lead to an altered germinal identity of the cells. However, no defects in differentiation, sperm quality, and fertility were observed in adult Tg Oct4-ERα36 animals tested (unpublished data). Nevertheless, the lack of defects in fertility after ERα36 overexpression in murine germ cells does not presuppose the lack of consequences in human germ cells. Conversely to murine germ cells, human germ cell differentiation is asynchronous, and the proliferative window is longer (several days in mice versus several months in humans).

#### 3.1.2. Impact of ERα36 on the Development of the Murine Mammary Gland

Estrogens play a major role during the post-pubertal development of the mammary gland by driving duct elongation and branching. Conversely, various knockout mouse studies indicate that the canonical estrogen receptors, ERα66 and ERβ1, are not necessary for pre-pubertal development of this organ [42,43]. Since ERα36 is a human-specific isoform, no information was so far available on its involvement during healthy mammary gland development. Thiebaut and colleagues [44] studied the impact of ERα36 expression using a MMTV-ERα36 transgenic C57BL/6J mouse model specifically expressing ERα36 in the mammary gland. To assess the impact of ERα36 expression on the embryonic and neonatal development, a quantitative analysis of the mammary tree and the histological aspect of the ducts were carried out in wt and Tg MMTV-ERα36 animals at weaning (21 days). The architecture of the rudimentary mammary tree, which develops during the embryonic and neonatal phase, was not affected by the expression of ERα36, whereas the epithelium appeared to be slightly thicker. In adulthood (16 weeks), the expression of ERα36 was associated with an altered mammary duct histology in virgin MMTV-ERα36 Tg mice. Indeed, there was a significant increase in the lumen and the stromal thickness as well as a significant decrease in the epithelium thickness and expression of the E-cadherin protein in the mammary epithelium. 

These observations suggested that, in response to endogenous estrogens produced at puberty, the activation of ERα36-dependent estrogen signaling altered the post-pubertal mammary duct histology. These data were reminiscent of those observed by Bocchinfuso and colleagues [42], who observed a hyperstimulation of the mammary estrogen response in wt mice treated with E_2_. The changes observed and especially the epithelium thinning and the loss of expression of E-cadherin suggested that ERα36 was involved in the control of proliferative and migratory abilities of mammary epithelial cells.

### 3.2. In Vitro and In Silico Studies on Healthy Breast Epithelial MCF-10A Cells

#### 3.2.1. Analysis of the Transcriptome of MCF-10A/ERα36 and MCF-10A/Zeo Cells

Thiebaut and colleagues [44], performed a transcriptomic analysis on MCF-10A/ERα36 and MCF-10A/Zeo cells. The main GO terms associated with ERa36 expression were “signal transduction”, “cell proliferation”, “cell surface receptor linked signal transduction”, and “apoptotic process” and the main signaling pathways were “PI3K/AkT signaling pathway”, “MAPK signaling pathway”, “cAMP signaling pathway”, and “JAK/STAT signaling pathway”. Interestingly, these biological processes and pathways are often described in the literature for their involvement in breast carcinogenesis [45]. In addition, some of these genes were previously described as markers associated with the epithelial-mesenchymal transition [46]. For instance, in MCF-10A/ERα36 cells, a significant decrease in the epithelial markers CDH1 and Ocln was observed, alongside a significant increase in the expression of the mesenchymal marker, CDH2. Moreover, a trend was observed for an increase in the expression of the mesenchymal marker Foxc2. Thereafter, the involvement of STAT3, NF-κB (p65), and PTEN in the ERα36-dependent signaling was validated in vitro, suggesting that the effects of ERα36 in healthy breast epithelial cells preferentially involved the NF-κB and JAK2/STAT3 signaling pathways.

#### 3.2.2. Phenotypic Study of MCF-10A/ERα36 and MCF-10A/Zeo Cells

In order to validate and clarify the data obtained in silico, Thiebaut and colleagues [44] explored the consequences of ERα36 overexpression on the proliferation, migration and apoptotic response of healthy breast epithelial MCF-10A cells. ERα36-dependent signaling was associated with resistance to apoptosis induced by staurosporine and an increase in the migratory potential of MCF-10A cells. Moreover, ERα36 overexpression led to a STAT3-dependent decrease in proliferation. Given that this factor is generally considered to be an inducer of proliferation, the role of ERβ in this process was then explored [47]. Indeed, this isoform or the estrogen receptor is mainly described in the literature for its antiproliferative effect on breast cancer cells [48], and a study by Wang and colleagues [49] showed that its expression is induced by STAT3 in pulmonary adenocarcinomas. ERα36 expression in MCF-10A cells led to a nuclear relocation of ERβ (Thiebaut, unpublished data) which appeared to be attenuated upon treatment with the STAT3 inhibitor, 5.15 DPP. This suggested a link between ERα36, STAT3, and ERβ, which could lead to a decrease in proliferation, triggered by ERα36 but dependent on ERβ activity.

These results obtained in vitro may provide insight into the histological aspect of the mammary epithelium observed in the Tg MMTV-ERα36 mouse model. On the one hand, it can be hypothesized that the increase in the epithelium thickness, which is observed at weaning in Tg MMTV-ERα36 animals, could mirror the resistance to apoptosis in breast epithelial cells expressing ERα36. Apoptosis plays a major role during morphogenesis by allowing the remodeling of new tissue structures. The disruption of this process could have an impact on the lumen width in the primary mammary tree. On the other hand, the decrease in epithelium thickness observed in adulthood could be due to slackening in cell division induced by ERα36 expression. Finally, the disorganization of the epithelium observed in adulthood could be linked to the loss of the epithelial identity (lower E-cadherin and occludin expression) of the duct cells or to acquired expression of mesenchymal markers (N-Cadherin) and migratory properties. The induction of migration and cell survival, which are two hallmarks of cancer cells, indicated that the expression of ERα36 played a role in the neoplastic transformation of the mammary epithelium. However, Chamard-Jovenin and colleagues [50] showed that nude mice grafted with MCF-10A/ERα36 or MCF-10A/Zeo did not develop tumors. This suggests that the sole overexpression of ERα36 in mice breast epithelial cells is not sufficient to induce tumor formation.

## 4. The Functions of ERα36 in Cancer

An increasing number of studies have clearly demonstrated that ERα36 plays a major role in tumor progression and in the development of resistance to cancer treatments. Nevertheless, conversely to ERα66, its expression is still not routinely addressed in clinics.

### 4.1. Mammary Tumors

Breast cancer (BC) is the most common cancer worldwide with the highest incidence and mortality rate in women. Recent data indicate that 2.1 million new cases of BC were diagnosed worldwide in 2018, i.e., approximately one quarter of all cancers diagnosed in women that year. The number of cancer-associated deaths that year was 630,000, which represents 15% of the all cancer-related deaths in women [51]. BC is a multifactorial disease resulting from a combination of factors from internal risks (gender, genetics, hormonal history) and external risk factors related to the environment (exposure to endocrine disruptive compounds, ionizing radiation…) and lifestyle (smoking, alcohol consumption, absence of physical activity, contraceptive or hormone replacement therapy intake…). The classification of mammary tumors reflects the heterogeneity of this pathology and is based on histological, anatomopathological, or molecular criteria. The molecular classification of BCs is based on the expression of 3 biomarkers: the estrogen receptor alpha (ERα66), the progesterone receptor (PR) and the human epidermal growth factor (HER2). This classification is mainly used to determine the prognosis and to guide the therapeutic strategy. Luminal A/B tumors (70% of total breast tumors) express one or both hormone receptors, are treated by hormone therapy (mainly the ER antagonist tamoxifen or the anti-aromatase anastrozole) and considered to be of good prognosis. HER2-positive tumors (10–15% of total breast tumors) are the target for effective anti-HER2 therapy (trastuzumab, lapatinib…). Lastly, tumors with no or little ER, PR, or HER2 expression are called “triple-negative” and have the worst prognosis, since patients are treated with high doses of radio/chemotherapy [51].

#### 4.1.1. The Prognostic Value of ERα36

The first retrospective study of 896 patients showed that 40% of the BCs expressed a high level of both the membrane and cytoplasmic forms of the ERα36 protein, independently of ERα expression. Moreover, its expression was associated with HER2 protein expression and with resistance to tamoxifen treatment [52]. Later, Wang and colleagues [53] confirmed that around 40% of patients express ERα36 at the plasma membrane, and although a high level of ERα36 expression was associated with tumor size and histological grade, they found no association with HER2 expression. In 2015, Chamard-Jovenin and colleagues [54] showed through a retrospective study of 118 breast tumor samples, that a strong expression of ERα36 mRNA was correlated with the expression of several metastatic markers. In addition, patients with ERα36-positive tumors were inclined to develop metastases and resistance to tamoxifen treatment [53]. Thus, from a clinical point of view, the high level of ERα36 expression in breast tumors was clearly associated with a poor prognosis due to enhanced metastatic potential of the tumor. A recent study confirmed that the expression of ERα36 was of poor prognosis in PR-positive tumors. However, this expression was significantly associated with longer survival in PR-negative tumors, suggesting a crosstalk between ERα36 and PR in breast cancer [55]. Therefore, ERα36 could become a new prognostic marker to discriminate a subset of PR tumors, generally associated with a good prognosis that may develop metastases.

#### 4.1.2. Molecular Mechanisms of Action

**Figure 4:**
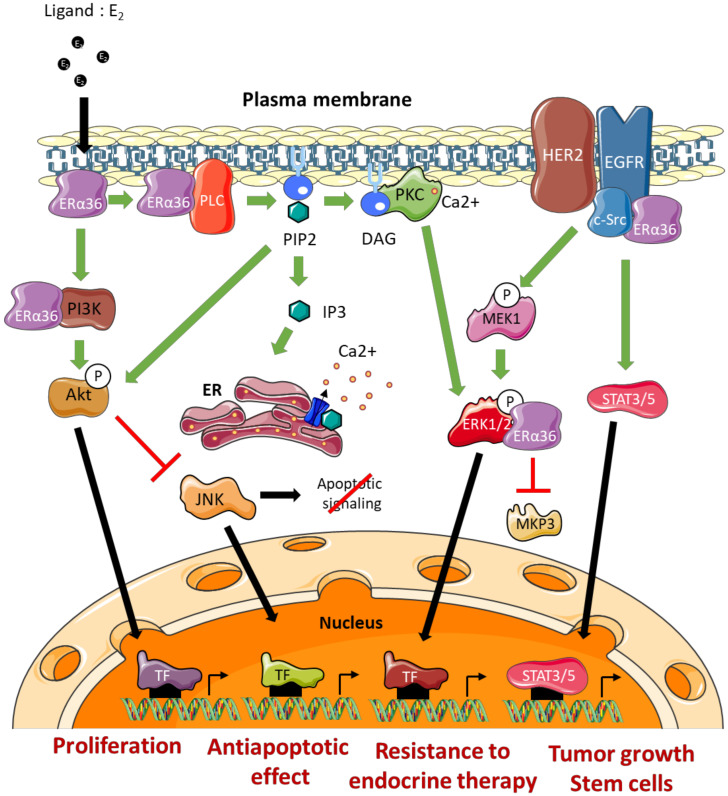
ERα36-dependent non genomic pathways. DAG: Diacylglycerol, ERK: Extracellular signal-Regulated Kinase, TF: Transcription Factor, IP3: Inositol trisphosphate, JNK: c-Jun N-terminal Kinase, MEK: MAP/ERK Kinase, MKP3: MAP Kinase Phosphatase 3, PIP2: Phosphatidylinositol-4,5-bisphosphate, PKC: Protein Kinase C, PLC: Phospholipase C, ER: Endoplasmic Reticulum.

The effects of ERα36 on mammary tumor progression and resistance to treatment are commonly linked to its ability to activate/mediate non-genomic signaling pathways (Figure 4). The impact of several ERα66 agonists (estrogens, estrogen-mimicking endocrine disruptors) and antagonists (tamoxifen or fulvestrant) on the modulation of ERα36 activity remains controversial [56]. Indeed, the LBD model of this variant, which was carried out based on the crystal structure of the LBD of the canonical form ERα66, suggests molecular interactions between ERα36 and the various potential ligands mentioned above. However, the binding affinity tests performed in vitro did not confirm the interactions predicted by the models. Nevertheless, these observations do not exclude that these ERα66 agonists and antagonists may indirectly modulate ERα36-dependent signaling or even the expression of ERα36 (see further). Moreover, Kang and colleagues [57] showed through other modeling studies and binding affinity tests that the GPER agonist, called G1, triggers a direct interaction and activation of ERα36.

##### The EGFR/Src/STAT3/5 Pathway

Studies by Zhang and colleagues [58,59] in several ERα66-positive breast cancer cell lines (MDA-MB-231 and MDA-MB-436) indicated that ERα36 could physically interact with Src. This interaction leads to the phosphorylation of Y416, associated with Src activation, as well as the dephosphorylation of Y527 which was associated with its inactivation. Once activated, Src was in turn responsible for activating EGFR via Y845 phosphorylation. The activation of this pathway leads to the induction of the STAT3/5 effector, which regulates the expression of cyclin D1 and thus stimulates tumor growth [60]. In connection with this pathway, it has also been shown that ERα36 could stabilize EGFR by preventing its degradation [58].

##### The ERα36/HER2/EGFR Pathway

A crosstalk between the signaling pathways induced by ERα36, HER2 and EGFR has been demonstrated and seems to play a major role in the acquisition of resistance to anti-estrogens. Indeed, Chu and colleagues [61] showed that the use of both tamoxifen and lapatinib (an EGFR inhibitor) restored the sensitivity of breast cancer MCF-7 cells to tamoxifen. In addition, this ERα36/EGFR/HER2 loop is involved in the development of resistance to chemotherapy, and more particularly to cisplatin, in MCF-7, BT474, and MDA-MB-231 breast cancer cells [62].

Finally, Kang and colleagues [63] demonstrated that ERα36 and HER2 cooperate for the maintenance and regulation of the population of ALDH1-positive breast cancer stem cells in tumors.

##### The Ras/MEK/ERK1/2 Pathway

In a model of HEK293 cells stably expressing ERα36, Wang and colleagues [11] showed that BSA-coupled E_2_, leads to ERα36 phosphorylation and triggers the activation of MEK1 and ERK1/2. In addition, tamoxifen or fulvestrant treatment of these cells promotes the ERα36-dependent phosphorylation of ERK1/2. These observations were confirmed in ERα66-positive MCF-7 cells that overexpress ERα36 and in ERα66-negative HCC38 cells which express this variant endogenously [11,64,65].

Recently, Omarjee and colleagues [30] demonstrated that ERα36 possesses a D domain which interacts with phosphorylated ERK2 (P-ERK2). This interaction stabilizes the activity of P-ERK2 by impeding the action of the phosphatase MKP3 which inactivates ERK2. P-ERK2 will in turn phosphorylate paxillin on Ser145 to activate the transcription of cyclin D1. In addition, ERα36 binding to P-ERK2 depends on the activation of Src [30].

##### The PKC-Dependent Pathway

The studies by Chaudhri and colleagues [65] conducted on HCC38 TNBC cells demonstrated the importance of the PKC-dependent pathway in ERα36-dependent signaling. Indeed, E_2_-BSA-dependent activation of PI3K/AkT/PKC by ERα36 leads to the activation of EMT metastatic factors, namely Snail1, CXCR4, and RANKL, concomitantly with E-cadherin loss of expression. Moreover, E_2_/ERα36 leads to the activation of PLC (Phospholipase C) and to the cleavage of PIP2 into IP3 and DAG [66]. IP3 triggers calcium release from the smooth endoplasmic reticulum, while DAG activated PKC, phosphorylates ERK1/2 to promote the proliferation and metastatic potential of tumor cells. In connection with this pathway, Chaudhri and colleagues [67] showed that PIP2 activated AkT and thus inhibited JNK (c-Jun Kinase), which initiates the apoptotic cascade. Therefore, ERα36-dependent signaling is also associated with the development of resistance to apoptosis mechanisms.

##### The PI3K/AkT Pathway

The activation of the PI3K/AkT pathway in connection with ERα36 was demonstrated in the context of Hec1A endometrial cancer cells. Indeed, in response to E_2_ or tamoxifen, a rapid activation of AkT leads to an increase in proliferation and an inhibition of the apoptotic signaling cascade [64,66,67]. In the presence of E_2_, ERα36 interacted directly with PI3K in the cytoplasm of TNBC cells (HBCc-12A line derived from HBCx-12A PDX). However, the mechanisms underlying this interaction have not yet been identified [30].

In conclusion, all of these results indicate that the ERα36 variant plays a pivotal role in various signaling pathways, previously described for their involvement in tumor progression. However, it is noteworthy that all of these data were obtained in vitro in different types of cell lines and mainly in models studying ERα36 overexpression. Their relevance remains to be addressed in vivo in a pathophysiological context. 

##### ERa36 and Other Hormonal Nuclear Receptors?

An emerging concept that hormone receptors crosstalk to regulate each other has emerged [68]. However, so far, ERα36 has been shown to regulate only ERα activity [11]. Recently, Konan and colleagues [55], showed that ERα36 binds to progesterone receptor (PR) and interferes with progesterone signaling. Indeed, ERα36 modulates PR expression, phosphorylation, and transcriptional activity. Interestingly, ERα36 is involved in the action of progesterone on cell proliferation and migration. Moreover, ERα36 expression is associated with a reduced metastasis disease-free survival in PR-positive tumors [55].

### 4.2. Other Cancers

The ERα36 variant has also been described for its role in the development and progression of other types of cancers.

#### 4.2.1. Endometrial Cancer

Although ERα36 expression is lower in cancer tissues than in normal endometrium, an increase in its expression is correlated with the progression and onset of metastases into the cervix [64]. Moreover, a strong expression of ERα36 is correlated with a significant decrease in disease-free survival (DFS) in endometrial cancer [34]. As in breast cancer, ERα36 protein localizes in cell membrane of hyperplastic endometrial cells and co-localizes with EGFR [69,70]. ERα36 stimulates proliferation of Hec1a endometrial cancer cells through the activation of MEK/ERK and PI3K/Akt pathways [64].

#### 4.2.2. Lung Adenocarcinoma

ERα36 expression is associated with poor prognosis in lung adenocarcinoma rather than in squamous cell carcinoma. In fact, Zhang and colleagues [71] revealed that in adenocarcinoma patients, high ERα-36 expression was associated with poorer DFS and overall survival (OS) whereas it is not the case for squamous cell carcinoma patients. High ERα36 expression was associated with lymph node metastasis in 92.3% of adenocarcinoma cases. Multivariate analysis on a cohort of 126 tumors showed that ERα36 was an independent prognostic marker for DFS [71].

#### 4.2.3. Renal Cell Carcinoma

Wang and colleagues [35] studied ERα36 expression by IHC in a cohort of 125 renal cancer cases. They showed that ERα36 expression positively correlated with tumor size, clinical stage, and necrosis. Moreover, patients with high ERα36 staining had a poor clinical outcome in terms of DFS and OS. This poor prognosis is amplified when ERα36 is localized at the membrane level. Multivariate analysis in this cohort show that ERα36 expression is an independent predictor of shorter DFS and that ERα36 localization is an independent prognostic marker for DFS and OS [35].

#### 4.2.4. Gastric Cancer

Although the expression of ERα36 is very high in several gastric cancer cell lines (SGC-7901, AGS, BGC-923, and MKN-45), two independent clinical studies regarding the expression of ERα36 in gastric cancer produced conflicting results. Indeed, in these two studies, the amount of ERα36 mRNA in cancer samples is alternately higher [72] or lower [33] than in normal tissue and ERα36 expression was not significantly associated with gender, age, grading, size of the tumor, or lymph node metastasis. However, ERα-36 expression positively correlated with Cyclin D1 and GRP94, two downstream effectors of ERα36-mediated non genomic estrogen signaling that could help to explain ERα36 function during gastric carcinogenesis [72,73].

#### 4.2.5. Colorectal Cancer

Conversely to most cancers, ERα36 seems to be associated with a good prognosis in colorectal cancer where its expression is inversely correlated with the rate of lymph node metastasis and tumor stage [74,75]. Jiang and colleagues [74] showed that ERα36 expression, which is correlated with that of ERα46, is downregulated in 71% of colorectal cancers tissues compared to the normal tissues.

## 5. How to Modulate ERα36 Expression or Activity?

### 5.1. Regulation of ERα36 Activity

The majority of *ESR1* gene mutations affecting the LBD and the activity of ERα66, are located in regions devoid of ERα36 [28]. Indeed, only amino acids 184–430 of ERα66 are conserved in the sequence of ERα36. Thus, we can hypothesize that the K303R, E380Q, and V392I mutations could have an impact on the activity of ERα36. Consistently, post-translational modifications, which affect the region 184 to 430 of ERα66, and in particular the phosphorylation of S282 and S305 residues, could also modulate ERα36 activity [28].

Moreover, the activity of the ERα36 variant can be regulated through chaperone proteins such as HSP90 or gamma synuclein. Unlike HSP90 which is also expressed under physiological conditions, gamma synuclein is expressed only in the context of breast tumors. The physical interaction between the gamma synuclein and ERα36, which is independent of the presence of HSP90, stimulates the activation of non-genomic signaling pathways, involving ERK1/2, in particular in response to E_2_ and tamoxifen [76]. Previous studies on three breast cancer cell lines (T47D, MCF-7, and MDA-MB-435) also showed that the expression of the gamma synuclein was associated with an increase in the transcriptional activity of ERα66. This suggested that this chaperone protein could also interact with ERα66 [77,78]. Thus, by stabilizing ERα36 in its active form, the gamma synuclein was associated with a poor prognosis as it participates in the induction of tumor progression (proliferation, migration) and in the development of resistance to tamoxifen [76,79].

### 5.2. Regulation ERα36 Expression

#### 5.2.1. Transcription Factors

Recently, Nagel and colleagues [80] studied the potential link between the amplification of the *ESR1* gene and the expression of ERα36, since the decrease in DFS associated with the expression of ERα36 in their cohort was similar to the decrease in DFS associated with the amplification of the *ESR1* gene as detected by qPCR. However, irrespective of the technique used, qPCR or FISH, no correlation could be established between the *ESR1* gene copy number and the level of ERα36 expression. It should be noted that none of the primers used targeted the ERα36-specific exon 9 of the *ESR1* gene [81].

**Figure 5:**
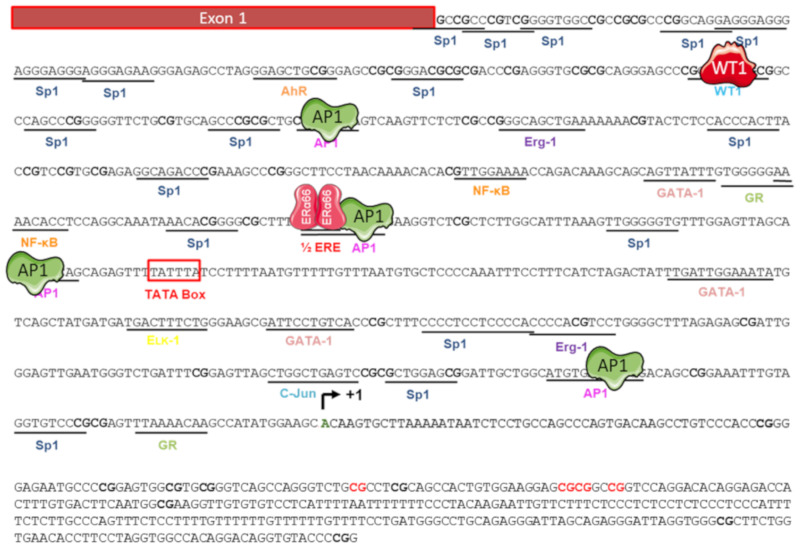
Regulators of the ERα36 promoter activity. The nucleotide sequence of the 5′ flanking region of ERα36 gene was cloned by Zou and colleagues in 2009 [82]. Putative binding sites of the transcription factors AP1, Sp1, AhR, NF-κB, and WT1, and an ERE half site, are underlined. The putative TATA box is shown as an open box. Various studies have demonstrated that activity of the ERα36 promoter is positively (green) regulated by AP1 but negatively (red) regulated by WT1 and the canonical form ERα66. The ERα36 transcription start site is indicated (curved arrow +1) in bold green. CpG dinucleotides are in bold and those whose level of methylation is correlated with ERα36 expression are indicated in bold red [84]. Adapted from [82,84].

The promoter region associated with the expression of ERα36 is located in the first intron of the *ESR1* gene and was cloned in 2009 by Zou and colleagues [82] (Figure 5). This region, which spans approximately 750 bp, is GC-rich and contains a non-canonical TATA box (−295 to −290), consensus sequences for the binding of several transcription factors. 

The presence of a half-ERE (5′-AGTCA-3′) in the ERα36 promoter suggests that its expression could be directly regulated by the canonical form ERα66. This was confirmed in HEK293 cells (ERα66-negative) by Zou and colleagues [82]. In addition, the inverse relationship between the expression of ERα66 and that of ERα36 is amplified by WT1 (Wilm’s Tumor 1) activity. Indeed, this factor positively regulates ERα66 expression while it negatively regulates that of ERα36 by binding to the 5’-GGGGCGCGCG-3’ consensus sequence located upstream of the TATA box [83].

To date, the direct link between ERα36 expression and the activity of transcription factors (TFs) binding to its promoter region has not been clearly described. However, some TFs interfere with estrogen signaling in BC. For instance, AhR activity was inversely correlated with the expression of ERα66 in a cohort of 439 patients and NF-κB exhibited greater activity in ERα66-negative and HER2-positive breast tumors [85,86]. In addition, a study by Vivacqua and colleagues [87] indicated that the expression of EGR-1 was induced in response to E_2_ and tamoxifen in ERα66-negative SKBr3 breast cancer cells in a GPER-EGFR-ERK-dependent manner. Finally, Kang and colleagues [57] also demonstrated that the GPER-dependent signaling stimulated the activity of the ERα36 promoter through Src/MEK1/2/AP-1 signaling.

Conversely, Yin and colleagues [88] showed that the expression of ERα36, EGFR, and HER2 was induced in response to tamoxifen in breast cancer MCF-7, T47D, and H3396 cells. This suggested the existence of a positive regulation loop between these three receptors. Indeed, the EGFR and HER2-dependent signaling was associated with an increase in ERα36 expression, through the AP-1 site located in the promoter region [63,88]. In turn, the induction of ERα36 expression is responsible for an increase in the level of HER2 and EGFR proteins.

Thus, despite these few scattered data, little information is available concerning the mechanisms, which regulate the expression of ERα36 in the context of breast cancer cells but also in a physiological context. However, such information could improve our understanding of the possible implication of ERα36 at the time of initiation and/or breast tumor progression, as well as during the acquisition of resistance to estrogen therapy.

#### 5.2.2. Epigenetic Regulation

Thiebaut and colleagues [84] identified four CpG islets located in the promoter region of ERα36. The methylation status of each of these islands was determined in 17 biopsies of breast tumors, indicating that the methylation of four CpG sites located in island four had a significant impact on the expression of ERα36, which increased upon methylation of these CpG sites. Although unexpected, this correlation between hypermethylation and the induction of gene expression is frequently observed when the CpG sites involved in regulation are located downstream of the TSS [89,90]. This direct link between the level of methylation of the ERα36 promoter and its expression level was confirmed in vitro by treating MCF-7 cells with a demethylating agent, decitabine (DAC). This treatment induced the demethylation of the four CpG sites previously identified and led to a decrease in ERα36 expression, without impacting cell viability, independently of ERα66 expression or activity (Figure 5). The transcriptional regulators capable of recognizing this differential methylation and thus of modulating the expression of ERα36 have yet to be identified.

#### 5.2.3. Post-Transcriptional Regulation of the Expression of ERα36 by microRNAs

Thiebaut and colleagues [84] identified 12 micro-RNAs that target ERα36 but not the 3′-UTR region of ERα66. They focused on hsa-miR-136-5p, the expression of which is two-fold lower in the serum of breast cancer patients than in healthy people. Moreover, the expression of this micro-RNA is inversely correlated with the stage/grade and the metastatic potential of tumors and mammary cell lines. Hsa-miR-136-5p is also described for its tumor suppressor role in the context of prostate cancer, renal carcinomas, pulmonary adenocarcinomas or osteosarcomas [91,92,93,94,95].

In this study, the authors demonstrated an inverse correlation between the expression of this micro-RNA and that of ERα36. In addition, they showed that hsa-miR-136-5p regulates the expression of this variant by interacting directly and specifically with the 3’-UTR region of its mRNA.

The hsa-miR-136-5p gene is located at the DLK1-DIO3 locus of chromosome 14q32 which includes one of the largest non-coding RNA clusters in the genome. Kagami and colleagues [96], showed that the expression of this locus is parentally imprinted and regulated by the level of methylation of 2 DMRs (Differentially Methylated Regions): IG-DMR and MEG3- DMR. Thiebaut et al. demonstrated that MCF-7 breast cancer cells exposed to DAC displayed an increase in hsa-miR-136-5p expression associated with a decrease in that of ERα36. They also showed that DAC blocks the 4-OHT-dependent induction of ERα36 expression in this cell line. Therefore, DAC, which has already been approved by the FDA for the treatment of myelodysplastic syndromes, could be used in combination with 4-OHT to cure ER-positive breast tumors and prevent ERα36-dependent acquired resistance to treatment.

#### 5.2.4. Indirect Modulators of Expression

In addition to estrogens, various molecules regulate the expression of ERα36. Anti-estrogens such as tamoxifen and fulvestrant stimulate ERα36 expression [11,53,88]. Other molecules such as flavonoids, i.e., broussoflavonol B and icaritin, purified respectively from the bark of the mulberry tree and a Chinese medicinal herb (Herba Epimedii), negatively regulate the level of expression of the ERα36 protein, through an unknown molecular mechanism. Both molecules are associated with an inhibition of the growth of breast cancer MDA-MB-231 cells by disrupting the positive regulatory loop with epidermal growth factor receptor (EGFR), which promotes malignant growth of TNBC cells [97,98]. E_2_-mediated ERα36 actions occur via E_2_ binding to the Glu 180 residue in helix 2 and the Arg 221 residue in helix 3 within ERα36. Furthermore, icaritin also binds to Glu 180 in helix 2, suggesting a competition between E_2_ and icaritin [97].

Another ERα36 down-regulator, called cyanidine-3-o-glucoside (Cy-3-glu), belongs to the family of anthocyanosides which are natural pigments of leaves, petals, and fruits. These are compounds known to inhibit the growth and metastatic potential of breast cancer cells. Cy-3-glu directly binds to the LBD of ERα36 and inhibits EGFR/Akt signaling in TNBC cells through EGFR degradation prior to inducing apoptosis [99].

Other studies, showed that a mixture of alkylphenols, named M4 (4-nonylphenol and 4-tert-octylphenol in ratio 30:1), representative of human exposure through commercially available foodstuffs [100], induced the expression of ERα36 in the seminoma cell line T-Cam2 and in healthy breast epithelial cells MCF-10A [39,44]. This mixture stimulated the proliferation, survival, and migration of both cell lines. Indeed, these estrogen-mimicking compounds are suspected of modulating ERα36-dependent signaling, namely the JAK/STAT pathway. 

Moreover, a transgenerational analysis of wt or MMTV-ERα36 Tg animals, exposed daily to M4 (0.5 µg/kg/day) throughout the gestation period was performed. At weaning (PND21), F3 wt mammary ducts showed an increased stroma and epithelium thickness together with a loss of E-cadherin expression. In Tg MMTV-ERα36 animals, the presence of the transgene seemed to counteract the effects associated with transgenerational exposure to M4. In adulthood, no change in mammary duct histology was observed in either wt or Tg animals MMTV-ERα36 [44,50].

A growing number of studies indicate that fetal or neonatal exposure to estrogen-mimetic endocrine disruptors, such as bisphenol A or vinclozoline, disrupts the physiological development of the mammary gland and could increase the risk of developing breast cancer in adulthood [101,102,103]. This suggests that early and/or hyperstimulation of estrogen signaling by endocrine disruptors may play a role in the mammary epithelium neoplastic transformation. Therefore, it remains to be determined if a continuous, early (fetal/neonatal) or late (post-pubertal) exposure to ERα36 modulators could affect the risk of developing breast cancer.

## 6. Future Prospects

While many publications now confirm the key role of ERα36 in neoplastic transformation and the progression of different types of solid tumors and begin to describe the mechanisms of regulation of its expression, these data have yet to be transformed into clinical applications.

**Figure 6:**
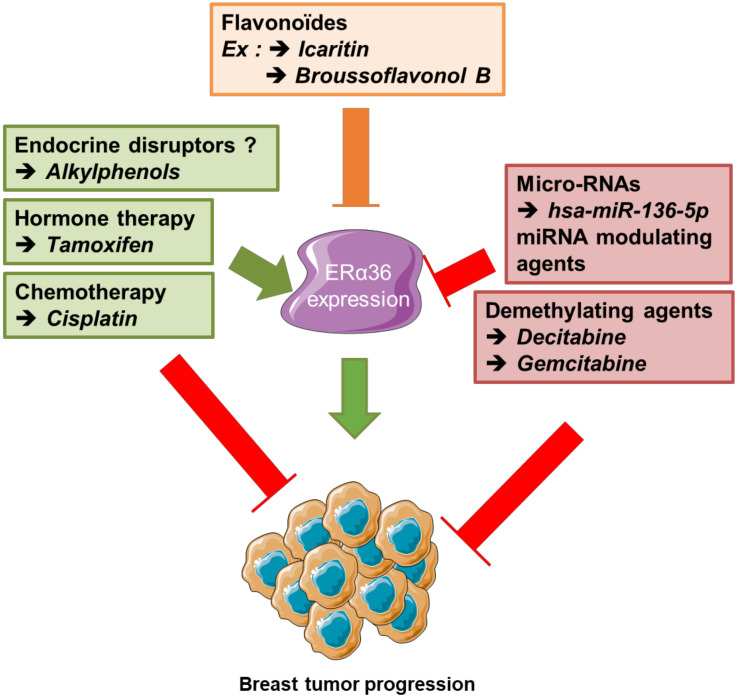
Summary of new therapeutic stategies, combined to counteract ERα36-dependent resistance to hormone therapy in breast cancer. Since ERα36 expression/ activity is stimulated by tamoxifen and cisplatin but down-regulated by broussoflavonol or Icaritin, decitabine and some micro-RNA overexpression, a dietary administration of flavonoids combined to anti-cancer strategy based on both hormone therapy and demethylating agents could prevent the emergence of ERα36-dependent resistance to hormone therapy in luminal breast tumors.

(i) To improve existing methods for classifying breast tumors by adding a molecular component related to the expression and cellular localization of ERα36. For this purpose, the use of “-omics” approaches coupled with bioinformatics appears to be highly promising.
(ii) To identify novel circulating biomarkers predictive of the expression level of ERα36 that could help improve diagnosis and patient follow-up. Indeed, it would be interesting to evaluate whether the detection of ERα36 mRNA or hsa-miR-136-5p in liquid biopsies from breast cancer patients could help to predict the evolution of the tumor and the fate of the patient. Indeed, a precocious increase in ERα36 mRNA and/or decrease in hsa-miR-136-5p in the plasma should be related to early resistance to hormone therapy (Figure 6).
(iii) To develop new therapeutic strategies targeting partners of ERα36 or ERα36-dependent signaling, for example in mammary tumors.

In approximately 60–70% of cases, breast tumors are hormone-dependent, as their growth and progression are controlled by estrogen and/or progesterone. In the context of ERα66-positive tumors, hormone therapy is often included in the therapeutic protocol, as a complement to therapeutic strategies based on surgery and chemotherapy. However, intrinsic or adaptive resistance to such treatments are observed in around 30% of cases and highlight the need for studies aimed at identifying molecular mechanisms that may decipher the mechanisms underlying these resistances.

Cohort studies conducted by Shi and colleagues [52] and Wang and colleagues [53], revealed that ERα36 expression in ERα66-positive breast tumors treated with tamoxifen is a poor prognosis. This deleterious effect appears to be largely related to the agonistic/ inducing effects of tamoxifen on ERα36 which lead to the activation of various signaling pathways involved in metastatic properties [53]. Indeed, treating MDA-MB 436 breast cancer cells with tamoxifen leads to the detection of an ERα36 modified heavy form of into the nucleus, which is associated with the induction the mammary cancer stem cell marker ALDH1 [53,104]. However, the mechanism underlying this ERα36-dependent nuclear localization and transcriptional regulation remains to be determined. Can ERα36 bind to other nuclear receptors or transcriptional factors to directly regulate target gene expression or serve as a nuclear transporter for other oncogenic transcription factors? Could this ERα36 nuclear localization be considered a new prognostic factor for cancer stem cell proliferation?

Finally, it seems that the ERα36/EGFR/HER2 loop plays an important role in tamoxifen resistance as Yin and colleagues showed that inhibition of this loop with a dual tyrosine kinase inhibitor, lapatinib, or ERα36 downregulator Broussoflavonol B restores the sensitivity of MCF-7 cells that were resistant to tamoxifen [88,105]. Furthermore, Icaritin and Cy-3-glu are associated with EGFR-dependent signaling inhibition and apoptosis of MDA-MB-231 triple negative breast cancer cells [97,99]. Therefore, the use of these inhibitors could counteract the positive ERα36/EGFR loop in the progression of triple-negative breast tumors or the ER36/EGFR/HER2 loop in tamoxifen resistance within ERα66–positive breast tumors.

These ERα36-dependent mechanisms are part of the acquired resistance to tamoxifen, while exposure to this anti-estrogen has been shown to be associated with an induction of ERα36 expression in MCF-7 breast cancer cells [88]. Similarly, Wang and colleagues [53] observed that ERα36 expression is significantly greater in tamoxifen-exposed breast tumors than in non-exposed tumors. Thiebaut et al. showed that co-exposure to 4-OHT and DAC counteracts tamoxifen-dependent induction of ERα36 expression in MCF-7 cells [84]. This combination of treatment could therefore restore the sensitivity of breast cancer cells to tamoxifen. As a continuum, it would be of interest to assess the combined effects of DAC and 4-OHT on breast cancer cell survival in vitro, but also in vivo in PDX tumor models resistant or not to tamoxifen [106].

So far, the link between ERα36 expression and the response of breast tumors to chemotherapy is not clearly established. Indeed, the studies by Shi and colleagues [52] and Wang and colleagues [53] have shown that the expression of this variant is not correlated with the survival of patients with ERα66-positive tumors treated with chemotherapy (cyclophosphamide or methotrexate). However, a study by Zhu and colleagues [62] showed that the variant ERα36 is involved in the development of resistance to cisplatin in MCF-7 cells and MDA-MB-231, depending on the ERα36/EGFR/HER2 loop. The involvement of ERα36 in the development of resistance to paclitaxel by MDA-MB-231 cells has also been demonstrated [59]. Interestingly, Thiebaut and colleagues [84] suggested that the ERα36 mRNA could be a target for hsa-miR-1253 microRNA, which is repressed in the paclitaxel-resistant Bads-200 and Bats-72 breast cancer cells [107]. Thus, it would be interesting to study the link between the expression of this microRNA and the expression of ERα36 in the context of acquired resistance to paclitaxel. Finally, it has also been shown that the JAK/STAT3/MAPK/Akt pathway, which is modulated by the expression of ERα36 [44], is involved in the resistance of MDA-MB 231 and MDA-MB 468 triple negative breast cancer cells to paclitaxel [108].

## 7. Conclusions

In conclusion, ERα36 now clearly appears as a tumor malignancy marker in many cancers and its high expression constitutes a reliable biomarker, predictive of disease progression and resistance to anti-tumor therapy. Furthermore, recent data from the literature provide new information on the regulation of its activity/expression, and propose new therapeutic approaches (epigenetic and/or medicinal) to modulate its expression and thus counteract metastatic progression and acquired resistance to treatment.

## References

[1] Hamilton K.J., Hewitt S.C., Arao Y., Korach K.S. (2017). Estrogen Hormone Biology. Curr. Top. Dev. Biol..

[2] Greene G.L., Gilna P., Waterfield M., Baker A., Hort Y., Shine J. (1986). Sequence and expression of human estrogen receptor complementary DNA. Science.

[3] Kuiper G.G., Enmark E., Pelto-Huikko M., Nilsson S., Gustafsson J.A. (1996). Cloning of a novel receptor expressed in rat prostate and ovary. Proc. Natl. Acad. Sci. USA.

[4] Menasce L.P., White G.R., Harrison C.J., Boyle J.M. (1993). Localization of the estrogen receptor locus (ESR) to chromosome 6q25.1 by FISH and a simple post-FISH banding technique. Genomics.

[5] Mosselman S., Polman J., Dijkema R. (1996). ER beta: Identification and characterization of a novel human estrogen receptor. FEBS Lett..

[6] Carmeci C., Thompson D.A., Ring H.Z., Francke U., Weigel R.J. (1997). Identification of a gene (GPR30) with homology to the G-protein-coupled receptor superfamily associated with estrogen receptor expression in breast cancer. Genomics.

[7] Revankar C.M., Cimino D.F., Sklar L.A., Arterburn J.B., Prossnitz E.R. (2005). A Transmembrane Intracellular Estrogen Receptor Mediates Rapid Cell Signaling. Science.

[8] Thomas P., Pang Y., Filardo E.J., Dong J. (2005). Identity of an Estrogen Membrane Receptor Coupled to a G Protein in Human Breast Cancer Cells. Endocrinology.

[9] Clarke C.H., Norfleet A.M., Clarke M.S., Watson C.S., Cunningham K.A., Thomas M.L. (2000). Perimembrane localization of the estrogen receptor alpha protein in neuronal processes of cultured hippocampal neurons. Neuroendocrinology.

[10] Li L., Haynes M.P., Bender J.R. (2003). Plasma membrane localization and function of the estrogen receptor alpha variant (ER46) in human endothelial cells. Proc. Natl. Acad. Sci. USA.

[11] Wang Z., Zhang X., Shen P., Loggie B.W., Chang Y., Deuel T.F. (2006). A variant of estrogen receptor-α, hER-α36: Transduction of estrogen- and antiestrogen-dependent membrane-initiated mitogenic signaling. Proc. Natl. Acad. Sci. USA.

[12] Gilad L.A., Schwartz B. (2007). Association of estrogen receptor β with plasma-membrane caveola components: Implication in control of vitamin D receptor. J. Mol. Endocrinol..

[13] Keaveney M., Klug J., Dawson M.T., Nestor P.V., Neilan J.G., Forde R.C., Gannon F. (1991). Evidence for a previously unidentified upstream exon in the human oestrogen receptor gene. J. Mol. Endocrinol..

[14] Kos M., Reid G., Denger S., Gannon F. (2001). Minireview: Genomic organization of the human ERalpha gene promoter region. Mol. Endocrinol..

[15] Wang Z., Zhang X., Shen P., Loggie B.W., Chang Y., Deuel T.F. (2005). Identification, cloning, and expression of human estrogen receptor-alpha36, a novel variant of human estrogen receptor-alpha66. Biochem. Biophys. Res. Commun..

[16] Gibson D.A., Saunders P.T.K. (2012). Estrogen dependent signaling in reproductive tissues—A role for estrogen receptors and estrogen related receptors. Mol. Cell. Endocrinol..

[17] Thornton J.W. (2001). Evolution of vertebrate steroid receptors from an ancestral estrogen receptor by ligand exploitation and serial genome expansions. Proc. Natl. Acad. Sci. USA.

[18] McEwan I.J. (2016). The Nuclear Receptor Superfamily at Thirty. Methods Mol. Biol. Clifton NJ.

[19] Ponglikitmongkol M., Green S., Chambon P. (1988). Genomic organization of the human oestrogen receptor gene. EMBO J..

[20] Kumar R., Thompson E.B. (1999). The structure of the nuclear hormone receptors. Steroids.

[21] Aranda A., Pascual A. (2001). Nuclear hormone receptors and gene expression. Physiol. Rev..

[22] Kumar R., Zakharov M.N., Khan S.H., Miki R., Jang H., Toraldo G., Singh R., Bhasin S., Jasuja R. (2011). The Dynamic Structure of the Estrogen Receptor. J. Amino Acids.

[23] Bourguet W., Germain P., Gronemeyer H. (2000). Nuclear receptor ligand-binding domains: Three-dimensional structures, molecular interactions and pharmacological implications. Trends Pharmacol. Sci..

[24] Heldring N., Pike A., Andersson S., Matthews J., Cheng G., Hartman J., Tujague M., Ström A., Treuter E., Warner M. (2007). Estrogen Receptors: How Do They Signal and What Are Their Targets. Physiol. Rev..

[25] Flouriot G., Brand H., Denger S., Metivier R., Kos M., Reid G., Sonntag-Buck V., Gannon F. (2000). Identification of a new isoform of the human estrogen receptor-alpha (hER-α) that is encoded by distinct transcripts and that is able to repress hER-α activation function 1. EMBO J..

[26] Chantalat E., Boudou F., Laurell H., Palierne G., Houtman R., Melchers D., Rochaix P., Filleron T., Stella A., Burlet-Schiltz O. (2016). The AF-1-deficient estrogen receptor ERα46 isoform is frequently expressed in human breast tumors. Breast Cancer Res..

[27] Zhu H., Huang Y., Su H., Ma Y., Tao Y., Liao D.J., Liu Y., Feng Z. (2018). Identification of a novel human estrogen receptor-α splice variant able to enhance malignant biological behaviors of breast cancer cells. Oncol. Lett..

[28] Le Romancer M., Poulard C., Cohen P., Sentis S., Renoir J.-M., Corbo L. (2011). Cracking the estrogen receptor’s posttranslational code in breast tumors. Endocr. Rev..

[29] Jia M., Dahlman-Wright K., Gustafsson J.-Å. (2015). Estrogen receptor alpha and beta in health and disease. Best Pract. Res. Clin. Endocrinol. Metab..

[30] Omarjee S., Jacquemetton J., Poulard C., Rochel N., Dejaegere A., Chebaro Y., Treilleux I., Marangoni E., Corbo L., Romancer M.L. (2017). The molecular mechanisms underlying the ERα-36-mediated signaling in breast cancer. Oncogene.

[31] Zheng Y., Zhang J., Xu Z., Sheng J., Zhang X., Wang H., Teng X., Liu X., Cao J., Teng L. (2010). Quantitative profiles of the mRNAs of ER-α and its novel variant ER-α36 in breast cancers and matched normal tissues. J. Zhejiang Univ. Sci. B.

[32] Xie H., Sun M., Liao X.-B., Yuan L.-Q., Sheng Z.-F., Meng J.-C., Wang D., Yu Z.-Y., Zhang L.-Y., Zhou H.-D. (2011). Estrogen receptor α36 mediates a bone-sparing effect of 17β-estrodiol in postmenopausal women. J. Bone Miner. Res..

[33] Wang J., Li J., Fang R., Xie S., Wang L., Xu C. (2012). Expression of ERα36 in gastric cancer samples and their matched normal tissues. Oncol. Lett..

[34] Sun L., Wang J., Zhang L., Li X., Shen D. (2013). Expression of ER-α36, a novel variant of estrogen receptor in endometrial carcinoma and its clinical significance. Gynecol. Obstet. Investig..

[35] Wang Q., Zhang W., Yang J., Liu Y.-L., Yan Z.-X., Guo Z.-J., Li Y.-J., Bian X.-W. (2015). High ERα36 Expression Level and Membrane Location Predict Poor Prognosis in Renal Cell Carcinoma. Medicine (Baltimore).

[36] Yan Y., Yu L., Castro L., Dixon D. (2017). ERα36, a variant of estrogen receptor α, is predominantly localized in mitochondria of human uterine smooth muscle and leiomyoma cells. PLoS ONE.

[37] Rajpert-De Meyts E. (2006). Developmental model for the pathogenesis of testicular carcinoma in situ: Genetic and environmental aspects. Hum. Reprod. Update.

[38] Fénichel P., Chevalier N. (2019). Is Testicular Germ Cell Cancer Estrogen Dependent? The Role of Endocrine Disrupting Chemicals. Endocrinology.

[39] Ajj H., Chesnel A., Pinel S., Plénat F., Flament S., Dumond H. (2013). An alkylphenol mix promotes seminoma derived cell proliferation through an ERalpha36-mediated mechanism. PLoS ONE.

[40] Yeom Y.I., Fuhrmann G., Ovitt C.E., Brehm A., Ohbo K., Gross M., Hübner K., Schöler H.R. (1996). Germline regulatory element of Oct-4 specific for the totipotent cycle of embryonal cells. Dev. Camb. Engl..

[41] Yoshimizu T., Sugiyama N., Felice M.D., Yeom Y.I., Ohbo K., Masuko K., Obinata M., Abe K., Schöler H.R., Matsui Y. (1999). Germline-specific expression of the Oct-4/green fluorescent protein (GFP) transgene in mice. Dev. Growth Differ..

[42] Bocchinfuso W.P., Lindzey J.K., Hewitt S.C., Clark J.A., Myers P.H., Cooper R., Korach K.S. (2000). Induction of Mammary Gland Development in Estrogen Receptor-α Knockout Mice. Endocrinology.

[43] Förster C., Mäkela S., Wärri A., Kietz S., Becker D., Hultenby K., Warner M., Gustafsson J.-Å. (2002). Involvement of estrogen receptor β in terminal differentiation of mammary gland epithelium. Proc. Natl. Acad. Sci. USA.

[44] Thiebaut C., Chamard-Jovenin C., Chesnel A., Morel C., Djermoune E.-H., Boukhobza T., Dumond H. (2017). Mammary epithelial cell phenotype disruption in vitro and in vivo through ERalpha36 overexpression. PLoS ONE.

[45] Velloso F., Bianco A., Farias J.O., Torres N.E., Ferruzo P.Y., Anschau V., Jesus-Ferreira H.C., Chang T.H.-T., Sogayar M., Zerbini L.F. The crossroads of breast cancer progression: Insights into the modulation of major signaling pathways. https://www.dovepress.com/the-crossroads-of-breast-cancer-progression-insights-into-the-modulati-peer-reviewed-fulltext-article-OTT.

[46] Asiedu M.K., Ingle J.N., Behrens M.D., Radisky D.C., Knutson K.L. (2011). TGFβ/TNFα-Mediated Epithelial-Mesenchymal Transition Generates Breast Cancer Stem Cells with a Claudin-Low Phenotype. Cancer Res..

[47] Banerjee K., Resat H. (2016). Constitutive activation of STAT3 in breast cancer cells: A review. Int. J. Cancer.

[48] Haldosén L.-A., Zhao C., Dahlman-Wright K. (2014). Estrogen receptor beta in breast cancer. Mol. Cell. Endocrinol..

[49] Wang H.-C., Yeh H.-H., Huang W.-L., Lin C.-C., Su W.-P., Chen H.H.W., Lai W.-W., Su W.-C. (2011). Activation of the signal transducer and activator of transcription 3 pathway up-regulates estrogen receptor-beta expression in lung adenocarcinoma cells. Mol. Endocrinol. Baltim. Md.

[50] Chamard-Jovenin C., Thiebaut C., Chesnel A., Bresso E., Morel C., Smail-Tabbone M., Devignes M.-D., Boukhobza T., Dumond H. (2017). Low-Dose Alkylphenol Exposure Promotes Mammary Epithelium Alterations and Transgenerational Developmental Defects, But Does Not Enhance Tumorigenic Behavior of Breast Cancer Cells. Front. Endocrinol..

[51] Bray F., Ferlay J., Soerjomataram I., Siegel R.L., Torre L.A., Jemal A. (2018). Global cancer statistics 2018: GLOBOCAN estimates of incidence and mortality worldwide for 36 cancers in 185 countries. CA Cancer J. Clin..

[52] Shi L., Dong B., Li Z., Lu Y., Ouyang T., Li J., Wang T., Fan Z., Fan T., Lin B. (2009). Expression of ER-α36, a Novel Variant of Estrogen Receptor α, and Resistance to Tamoxifen Treatment in Breast Cancer. J. Clin. Oncol..

[53] Wang Q., Jiang J., Ying G., Xie X.-Q., Zhang X., Xu W., Zhang X., Song E., Bu H., Ping Y.-F. (2018). Tamoxifen enhances stemness and promotes metastasis of ERα36+ breast cancer by upregulating ALDH1A1 in cancer cells. Cell Res..

[54] Chamard-Jovenin C., Jung A.C., Chesnel A., Abecassis J., Flament S., Ledrappier S., Macabre C., Boukhobza T., Dumond H. (2015). From ERα66 to ERα36: A generic method for validating a prognosis marker of breast tumor progression. BMC Syst. Biol..

[55] Konan H., Kassem L., Omarjee S., Surmieliova-Garnes A., Jacquemetton J., Cascales E., Rezza A., Trédan O., Treilleux I., Poulard C. (2020). ERα-36 regulates progesterone signaling in breast cancer. Breast Cancer Res..

[56] Lin A.H.Y., Li R.W.S., Ho E.Y.W., Leung G.P.H., Leung S.W.S., Vanhoutte P.M., Man R.Y.K. (2013). Differential Ligand Binding Affinities of Human Estrogen Receptor-α Isoforms. PLoS ONE.

[57] Kang L., Zhang X., Xie Y., Tu Y., Wang D., Liu Z., Wang Z.-Y. (2010). Involvement of Estrogen Receptor Variant ER-α36, Not GPR30, in Nongenomic Estrogen Signaling. Mol. Endocrinol..

[58] Zhang X., Kang L., Ding L., Vranic S., Gatalica Z., Wang Z.-Y. (2011). A Positive Feedback Loop of ER-α36/EGFR Promotes Malignant Growth of ER-negative Breast Cancer Cells. Oncogene.

[59] Zhang J., Li G., Li Z., Yu X., Zheng Y., Jin K., Wang H., Gong Y., Sun X., Teng X. (2012). Estrogen-independent effects of ER-α36 in ER-negative breast cancer. Steroids.

[60] Rao J., Jiang X., Wang Y., Chen B. (2011). Advances in the understanding of the structure and function of ER-α36,a novel variant of human estrogen receptor-alpha. J. Steroid Biochem. Mol. Biol..

[61] Chu I., Blackwell K., Chen S., Slingerland J. (2005). The dual ErbB1/ErbB2 inhibitor, lapatinib (GW572016), cooperates with tamoxifen to inhibit both cell proliferation- and estrogen-dependent gene expression in antiestrogen-resistant breast cancer. Cancer Res..

[62] Zhu L., Zou J., Zhao Y., Jiang X., Wang Y., Wang X., Chen B. (2018). ER-α36 mediates cisplatin resistance in breast cancer cells through EGFR/HER-2/ERK signaling pathway. J. Exp. Clin. Cancer Res. CR.

[63] Kang L., Guo Y., Zhang X., Meng J., Wang Z.-Y. (2011). A positive cross-regulation of HER2 and ER-α36 controls ALDH1 positive breast cancer cells. J. Steroid Biochem. Mol. Biol..

[64] Lin S.-L., Yan L.-Y., Zhang X.-T., Yuan J., Li M., Qiao J., Wang Z.-Y., Sun Q.-Y. (2010). ER-α36, a Variant of ER-α, Promotes Tamoxifen Agonist Action in Endometrial Cancer Cells via the MAPK/ERK and PI3K/Akt Pathways. PLoS ONE.

[65] Chaudhri R.A., Olivares-Navarrete R., Cuenca N., Hadadi A., Boyan B.D., Schwartz Z. (2012). Membrane estrogen signaling enhances tumorigenesis and metastatic potential of breast cancer cells via estrogen receptor-α36 (ERα36). J. Biol. Chem..

[66] Chaudhri R.A., Schwartz N., Elbaradie K., Schwartz Z., Boyan B.D. (2014). Role of ERα36 in membrane-associated signaling by estrogen. Steroids.

[67] Chaudhri R.A., Hadadi A., Lobachev K.S., Schwartz Z., Boyan B.D. (2014). Estrogen receptor-alpha 36 mediates the anti-apoptotic effect of estradiol in triple negative breast cancer cells via a membrane-associated mechanism. Biochim. Biophys. Acta.

[68] Truong T.H., Dwyer A.R., Diep C.H., Hu H., Hagen K.M., Lange C.A. (2019). Phosphorylated Progesterone Receptor Isoforms Mediate Opposing Stem Cell and Proliferative Breast Cancer Cell Fates. Endocrinology.

[69] Tu B.-B., Lin S.-L., Yan L.-Y., Wang Z.-Y., Sun Q.-Y., Qiao J. (2011). ER-α36, a novel variant of estrogen receptor α, is involved in EGFR-related carcinogenesis in endometrial cancer. Am. J. Obstet. Gynecol..

[70] Lin S.-L., Yan L.-Y., Liang X.-W., Wang Z.-B., Wang Z.-Y., Qiao J., Schatten H., Sun Q.-Y. (2009). A novel variant of ER-alpha, ER-alpha36 mediates testosterone-stimulated ERK and Akt activation in endometrial cancer Hec1A cells. Reprod. Biol. Endocrinol. RBE.

[71] Zhang S., Qiu C., Wang L., Liu Q., Du J. (2014). The elevated level of ERα36 is correlated with nodal metastasis and poor prognosis in lung adenocarcinoma. Steroids.

[72] Deng H., Huang X., Fan J., Wang L., Xia Q., Yang X., Wang Z., Liu L. (2010). A variant of estrogen receptor-α, ER-α36 is expressed in human gastric cancer and is highly correlated with lymph node metastasis. Oncol. Rep..

[73] Fu Z., Zhen H., Zou F., Wang X., Chen Y., Liu L. (2014). Involvement of the Akt signaling pathway in ER-α36/GRP94-mediated signaling in gastric cancer. Oncol. Lett..

[74] Jiang H., Teng R., Wang Q., Zhang X., Wang H., Wang Z., Cao J., Teng L. (2008). Transcriptional analysis of estrogen receptor alpha variant mRNAs in colorectal cancers and their matched normal colorectal tissues. J. Steroid Biochem. Mol. Biol..

[75] Sołtysik K., Czekaj P. (2015). ERα36—Another piece of the estrogen puzzle. Eur. J. Cell Biol..

[76] Shi Y.E., Chen Y., Dackour R., Potters L., Wang S., Ding Q., Wang Z., Liu Y.E. (2010). Synuclein gamma stimulates membrane-initiated estrogen signaling by chaperoning estrogen receptor (ER)-alpha36, a variant of ER-alpha. Am. J. Pathol..

[77] Jiang Y., Liu Y.E., Lu A., Gupta A., Goldberg I.D., Liu J., Shi Y.E. (2003). Stimulation of estrogen receptor signaling by gamma synuclein. Cancer Res..

[78] Liu Y.E., Pu W., Jiang Y., Shi D., Dackour R., Shi Y.E. (2007). Chaperoning of estrogen receptor and induction of mammary gland proliferation by neuronal protein synuclein gamma. Oncogene.

[79] Panneerselvam M., Muthu K., Ramadas K. (2015). Structural insights into tumor-specific chaperoning activity of gamma synuclein in protecting estrogen receptor alpha 36 and its role in tamoxifen resistance in breast cancer. Mol. Biosyst..

[80] Nagel A., Szade J., Iliszko M., Elzanowska J., Welnicka-Jaskiewicz M., Skokowski J., Stasilojc G., Bigda J., Sadej R., Zaczek A. (2019). Clinical and Biological Significance of ESR1 Gene Alteration and Estrogen Receptors Isoforms Expression in Breast Cancer Patients. Int. J. Mol. Sci..

[81] Markiewicz A., Wełnicka-Jaśkiewicz M., Skokowski J., Jaśkiewicz J., Szade J., Jassem J., Zaczek A.J. (2013). Prognostic significance of ESR1 amplification and ESR1 PvuII, CYP2C19*2, UGT2B15*2 polymorphisms in breast cancer patients. PLoS ONE.

[82] Zou Y., Ding L., Coleman M., Wang Z. (2009). Estrogen receptor-alpha (ER-α) suppresses expression of its variant ER-α36. FEBS Lett..

[83] Kang L., Wang L., Wang Z.-Y. (2011). Opposite regulation of estrogen receptor-α and its variant ER-α36 by the Wilms’ tumor suppressor WT1. Oncol. Lett..

[84] Thiebaut C., Chesnel A., Merlin J.-L., Chesnel M., Leroux A., Harlé A., Dumond H. (2019). Dual Epigenetic Regulation of ERα36 Expression in Breast Cancer Cells. Int. J. Mol. Sci..

[85] Biswas D.K., Cruz A.P., Gansberger E., Pardee A.B. (2000). Epidermal growth factor-induced nuclear factor kappa B activation: A major pathway of cell-cycle progression in estrogen-receptor negative breast cancer cells. Proc. Natl. Acad. Sci. USA.

[86] Vacher S., Castagnet P., Chemlali W., Lallemand F., Meseure D., Pocard M., Bieche I., Perrot-Applanat M. (2018). High AHR expression in breast tumors correlates with expression of genes from several signaling pathways namely inflammation and endogenous tryptophan metabolism. PLoS ONE.

[87] Vivacqua A., Romeo E., De Marco P., De Francesco E.M., Abonante S., Maggiolini M. (2012). GPER mediates the Egr-1 expression induced by 17β-estradiol and 4-hydroxitamoxifen in breast and endometrial cancer cells. Breast Cancer Res. Treat..

[88] Yin L., Zhang X.-T., Bian X.-W., Guo Y.-M., Wang Z.-Y. (2014). Disruption of the ER-α36-EGFR/HER2 positive regulatory loops restores tamoxifen sensitivity in tamoxifen resistance breast cancer cells. PLoS ONE.

[89] Ball M.P., Li J.B., Gao Y., Lee J.-H., LeProust E., Park I.-H., Xie B., Daley G.Q., Church G.M. (2009). Targeted and genome-scale methylomics reveals gene body signatures in human cell lines. Nat. Biotechnol..

[90] Ehrlich M., Lacey M. (2013). DNA methylation and differentiation: Silencing, upregulation and modulation of gene expression. Epigenomics.

[91] Chen P., Zhao L., Pan X., Jin L., Lin C., Xu W., Xu J., Guan X., Wu X., Wang Y. (2018). Tumor suppressor microRNA-136-5p regulates the cellular function of renal cell carcinoma. Oncol. Lett..

[92] Guo T., Pan G. (2018). MicroRNA-136 functions as a tumor suppressor in osteosarcoma via regulating metadherin. Cancer Biomark. Sect. Dis. Markers.

[93] Li T.-T., Gao X., Gao L., Gan B.-L., Xie Z.-C., Zeng J.-J., Chen G. (2018). Role of upregulated miR-136-5p in lung adenocarcinoma: A study of 1242 samples utilizing bioinformatics analysis. Pathol. Res. Pract..

[94] Xie Z.-C., Li T.-T., Gan B.-L., Gao X., Gao L., Chen G., Hu X.-H. (2018). Investigation of miR-136-5p key target genes and pathways in lung squamous cell cancer based on TCGA database and bioinformatics analysis. Pathol. Res. Pract..

[95] Zhu Y., Shao S., Pan H., Cheng Z., Rui X. (2018). MicroRNA-136 inhibits prostate cancer cell proliferation and invasion by directly targeting mitogen-activated protein kinase kinase 4. Mol. Med. Rep..

[96] Kagami M., O’Sullivan M.J., Green A.J., Watabe Y., Arisaka O., Masawa N., Matsuoka K., Fukami M., Matsubara K., Kato F. (2010). The IG-DMR and the MEG3-DMR at Human Chromosome 14q32.2: Hierarchical Interaction and Distinct Functional Properties as Imprinting Control Centers. PLoS Genet..

[97] Wang X., Zheng N., Dong J., Wang X., Liu L., Huang J. (2017). Estrogen receptor-α36 is involved in icaritin induced growth inhibition of triple-negative breast cancer cells. J. Steroid Biochem. Mol. Biol..

[98] Guo M., Wang M., Deng H., Zhang X., Wang Z.-Y. (2013). A novel anticancer agent Broussoflavonol B downregulates estrogen receptor (ER)-α36 expression and inhibits growth of ER-negative breast cancer MDA-MB-231 cells. Eur. J. Pharmacol..

[99] Wang L., Li H., Yang S., Ma W., Liu M., Guo S., Zhan J., Zhang H., Tsang S.Y., Zhang Z. (2016). Cyanidin-3-o-glucoside directly binds to ERα36 and inhibits EGFR-positive triple-negative breast cancer. Oncotarget.

[100] Raecker T., Thiele B., Boehme R.M., Guenther K. (2011). Endocrine disrupting nonyl- and octylphenol in infant food in Germany: Considerable daily intake of nonylphenol for babies. Chemosphere.

[101] Nilsson E.E., Anway M.D., Stanfield J., Skinner M.K. (2008). Transgenerational epigenetic effects of the endocrine disruptor vinclozolin on pregnancies and female adult onset disease. Reprod. Camb. Engl..

[102] Manikkam M., Guerrero-Bosagna C., Tracey R., Haque M.M., Skinner M.K. (2012). Transgenerational Actions of Environmental Compounds on Reproductive Disease and Identification of Epigenetic Biomarkers of Ancestral Exposures. PLoS ONE.

[103] Dhimolea E., Wadia P.R., Murray T.J., Settles M.L., Treitman J.D., Sonnenschein C., Shioda T., Soto A.M. (2014). Prenatal Exposure to BPA Alters the Epigenome of the Rat Mammary Gland and Increases the Propensity to Neoplastic Development. PLoS ONE.

[104] Burness M.L., Wicha M.S. Tamoxifen and ERα36: Fertilizing the seeds of breast cancer metastasis. http://www.nature.com/articles/s41422-018-0028-4.

[105] Teymourzadeh A., Mansouri S., Farahmand L., Hosseinzade A., Majidzadeh-A K. (2017). ER-α36 Interactions With Cytosolic Molecular Network in Acquired Tamoxifen Resistance. Clin. Breast Cancer.

[106] Poulard C., Jacquemetton J., Trédan O., Cohen P.A., Vendrell J., Ghayad S.E., Treilleux I., Marangoni E., Le Romancer M. (2019). Oestrogen Non-Genomic Signalling is Activated in Tamoxifen-Resistant Breast Cancer. Int. J. Mol. Sci..

[107] Lou W., Liu J., Ding B., Xu L., Fan W. (2018). Identification of chemoresistance-associated miRNAs in breast cancer. Cancer Manag. Res..

[108] Wang S., Yao Y., Yao M., Fu P., Wang W. (2018). Interleukin-22 promotes triple negative breast cancer cells migration and paclitaxel resistance through JAK-STAT3/MAPKs/AKT signaling pathways. Biochem. Biophys. Res. Commun..

